# Increased Angiogenesis and Lymphangiogenesis in the Placental Villi of Women with Chronic Venous Disease during Pregnancy

**DOI:** 10.3390/ijms21072487

**Published:** 2020-04-03

**Authors:** Miguel A Ortega, Miguel A Saez, Oscar Fraile-Martínez, Ángel Asúnsolo, Leonel Pekarek, Coral Bravo, Santiago Coca, Felipe Sainz, Melchor Álvarez- Mon, Julia Buján, Natalio García-Honduvilla

**Affiliations:** 1 Department of Medicine and Medical Specialties, Faculty of Medicine and Health Sciences and Networking Biomedical Research Centre on Bioengineering, Biomaterials and Nanomedicine (CIBER-BBN), University of Alcalá, 28801 Alcalá de Henares, Madrid, Spain; miguel.angel.ortega92@gmail.com (M.A.O.); msaega1@oc.mde.es (M.A.S.); oscarfra.7@hotmail.com (O.F.-M.); leonel.pekarek@gmail.com (L.P.); s.coca@uah.es (S.C.); natalio.garcia@uah.es (N.G.-H.); 2 Ramón y Cajal Institute of Healthcare Research (IRYCIS), 28034 Madrid, Spain; angel.asunsolo@uah.es; 3 Pathological Anatomy Service, Central University Hospital of Defence-UAH Madrid, 28801 Alcalá de Henares, Madrid, Spain; 4 Department of Surgery, Medical and Social Sciences, Faculty of Medicine and Health Sciences, University of Alcalá, 28801 Alcalá de Henares, Madrid, Spain; cbravoarribas@gmail.com (C.B.); sainz.felipe@gmail.com (F.S.); 5 Service of Gynecology and Obstetrics, Section of Fetal Maternal Medicine, Central University Hospital of Defence-UAH Madrid, 28801 Alcalá de Henares, Madrid, Spain; 6 Angiology and Vascular Surgery Service, Central University Hospital of Defence-UAH Madrid, 28801 Alcalá de Henares, Madrid, Spain; 7 Immune System Diseases-Rheumatology and Oncology Service, University Hospital Príncipe de Asturias, CIBEREHD, 28801 Alcalá de Henares, Madrid, Spain

**Keywords:** chronic venous disease, pregnancy, CD31, podoplanin (D2-40), Flt-1, PIGF

## Abstract

Pregnancy is a period in a woman’s life associated with an increased risk of developing lower extremity chronic venous disease (CVD). Pregnancy-associated CVD is associated with changes in placental villi. We investigated angiogenesis and lymphangiogenesis in the placental villi of women with CVD during pregnancy compared with healthy controls with no history of CVD (HC). An observational, analytical, and prospective cohort study was conducted on 114 women in their third trimester of pregnancy (32 weeks). Sixty-two participants were clinically diagnosed with CVD. In parallel, 52 controls with no history of CVD (HC) were studied. Gene and protein expression of CD31, podoplanin (D2-40), Flt-1, and placental growth factor (PIGF) was analysed by real-time polymerase chain reaction (RT-qPCR) and immunohistochemistry. CD31 and D2-40 gene expression was significantly greater in the placental villi of women with CVD, as were the numbers of vessels positive for CD31 and D2-40. Significantly higher gene and protein expression of Flt-1 and PIGF was observed in the placental villi of women with CVD. Histological analysis showed more placental villi with periodic acid of Schiff (PAS)-positive material in women with CVD. Our results show a connection between pregnancy-associated CVD and leading to higher proangiogenic and lymphangiogenic activity in placental villi.

## 1. Introduction

Chronic venous disease (CVD) refers to various anomalies commonly affecting the lower extremities, in which venous return is abnormal, and varicose veins are the most important clinical manifestation [1]. Pregnancy is associated with an increased risk of developing chronic venous disorder [2,3]. Risk factors associated with venous disorder during pregnancy include genetic factors and family history, occupation, and the number of pregnancies or the interval between pregnancies [4]. Several haemodynamic changes occur in pregnant women, including greater vasodilation, smooth muscle cell relaxation, compression of the iliac veins due to increased uterine size, slower venous flow, stasis, and venous valve incompetence. All this results in a situation of venous hypertension, which can lead to the development of varicose veins, especially during the third trimester of pregnancy [5,6,7]. Both oestrogen and progesterone play an important role in all these changes in the cardiovascular system, and especially in CVD [8,9].

The roles of all these changes in the placenta have not yet been fully elucidated. We have previously shown that lower extremity CVD during pregnancy is associated with some placental changes, such as extracellular matrix remodelling or increased placental apoptosis and villous calcification, along with increased expression of tissue hypoxia markers [10,11,12]. These mechanisms are also dysregulated at the placental level in other important vascular diseases, such as preeclampsia and intrauterine foetal growth restriction [13,14,15]. It should be highlighted how all these pathological conditions affect the process of angiogenesis. Placental growth factor (PIGF) has an important role in angiogenesis, as well as in the proper function of the placenta [16,17]. In women with CVD during pregnancy, overexpression of vascular endothelial growth factor (VEGF) has been reported, explaining its role in the disease’s pathogenesis [12]. Likewise, the VEGF receptors VEGFR-1 (Flt-1) and VEGFR-2 (KDR/Flk-2) play roles in various vascular diseases, such as preeclampsia [18,19]. Interestingly, VEGF has been observed to regulate, through Flt-1 and KDR signalling, the levels of sFlt-1 a soluble VEGF and PIGF receptor overexpressed in such pregnancy vascular diseases, showing the importance of the balance of these factors during pregnancy [20,21,22]. On the basis of the above, the aim of the present study is to examine angiogenesis and lymphogenesis in the placental villi of women with CVD during pregnancy compared with patients without CVD, by examining the gene and protein expression of CD31, podoplanin (D2-40), Flt-1, and PIGF.

## 2. Results

### 2.1. More Vessels Positive for CD31 and D2-40 in the Placental Villi of Women with CVD

**Figure 1:**
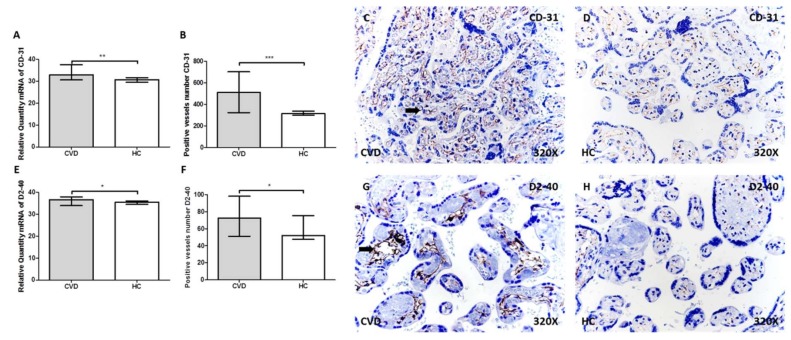
**A–B.** mRNA expression levels by RT-qPCR and positive vessels number for CD-31. **C–D.** Images showing the immunostaining for CD-31 in the placental villi for CVD (C) and HC (D) by immunohistochemistry. **E–F.** mRNA expression levels by RT-qPCR and positive vessels number for D2-40. **G–H.** Images showing the immunostaining for D2-40 in the placental villi for CVD (G) and HC (H) by immunohistochemistry. CVD = pregnancy-associated lower extremity chronic venous disease. HC = healthy controls with no history of CVD. *p* < 0.05 (*), *p* < 0.01 (**), and *p* < 0.001 (***). Arrow = positive protein expression.

We studied specific markers of angiogenesis (CD31) and lymphangiogenesis (D2-40) in the placental villi of women with CVD versus healthy controls (HCs), using RT-qPCR and immunohistochemistry. CD31 gene expression was significantly higher in placental villi of women with CVD (CVD = 32.83 (30.66–37.59), HC = 30.71 (27.51–33.77), ** *p* = 0.0019, Figure 1A). The number of vessels with positive CD31 protein expression was significantly higher in CVD than HC (CVD = 498.00 (324.00–703.00), HC = 315.00 (275.00–578.00), *** *p* < 0.0001, Figure 1B). The images in Figure 1C,D show CD31 was present in the capillary endothelium of placental villi of both study groups, with more intensity in the CVD group (arrow).

Significantly higher D2-40 (podoplanin) gene expression was seen in placental villi of the CVD group (CVD = 37.01 (33.95–38.05), HC = 35.53 (33.62–38.41), * *p* = 0.0243, Figure 1E). The number of vessels positive for D2-40 protein expression was significantly higher in placental villi of the CVD group compared with HC (CVD = 78.50 (51.00–98.00), HC = 52.00 (42.00–86.00), * *p* = 0.0104, Figure 1F). D2-40 was distributed sinuously along placental villi, being more intense in the CVD group (Figure 1G,H, arrow).

### 2.2. Women with CVD Show Greater Expression of Flt-1 and PIGF in Placental Villi

**Figure 2:**
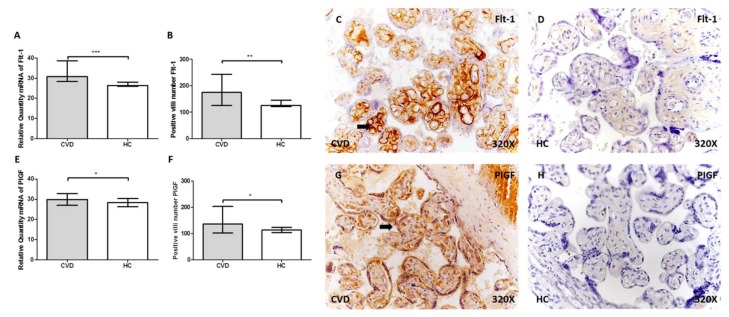
**A–B.** mRNA expression levels by RT-qPCR and positive vessels number for Flt-1. **C–D.** Images showing the immunostaining for Flt-1 in the placental villi for CVD (**C**) and HC (**D**) by immunohistochemistry. **E–F.** mRNA expression levels by RT-qPCR and positive vessels number for placental growth factor (PIGF). **G–H.** Images showing the immunostaining for PIGF in the placental villi for CVD (**G**) and HC (**H**) by immunohistochemistry. CVD = pregnancy-associated lower extremity chronic venous disease. HC = healthy controls with no history of CVD. *p* < 0.05 (*), *p* < 0.01 (**), and *p* < 0.001 (***). Arrow = positive protein expression.

Flt-1 gene expression was significantly higher in placental villi of CVD than HC (CVD = 29.59 (28.35–38.60), HC = 26.36 (24.45–29.46), *** *p* < 0.0001, Figure 2A). The CVD group had more positive villi for Flt-1 (CVD = 167.00 (125.00–243.00), HC = 126.00 (104.00–196.00), ** *p* = 0.012, Figure 2B). Flt-1 was distributed homogeneously in the extracellular matrix of placental villi of the study groups, with more intense expression in CVD (Figure 2C,D, arrow).

PIGF gene expression was significantly greater in placental villi of women with CVD (CVD = 30.07 (26.96–32.80), HC = 28.27 (24.66–30.79), * *p* = 0.036, Figure 2E), and these women had more placental villi positive for PIGF (CVD = 132.00 (102.00–203.00), HC = 113.00 (99.00–165.00), * *p* = 0.0224, Figure 2F). PIGF was found in the syncytiotrophoblast, cytotrophoblast, and extracellular matrix of placental villi, with more intensity in the CVD group (Figure 2G,H, arrow).

### 2.3. More Placental Villi with Periodic Acid of Schiff (PAS)-Positive Material in Women with CVD

**Figure 3:**
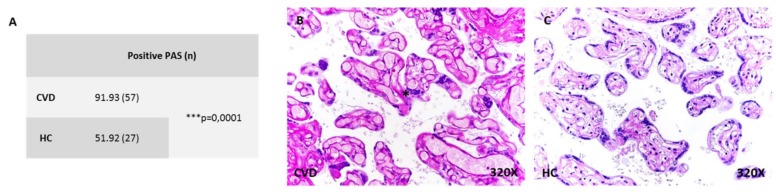
**A.** Percentage of positive periodic acid of Schiff (PAS) placental villi in CVD and HC. **B–C.** Representative images of PAS in CVD (**B**) and HC (**C**). CVD = pregnancy-associated lower extremity chronic venous disease. HC = healthy controls with no history of CVD. *p* < 0.001 (***). Asterisk = positive reaction.

PAS staining showed that the percentage of placentas with PAS-positive material in villi was 91.93% (n = 57) in the CVD group, while it was 51.92% (n = 27) in the HC group, (Figure 3A). Placental villi in CVD showed greater staining with a more intense leucofuchsin reaction (Figure 3B,C). The Pearson χ² test showed a significance of *** *p* < 0.0001.

## 3. Discussion

Pregnancy causes haemodynamic and mechanical changes, triggering vascular complications such as lower extremity CVD [23]. Our previous studies showed how impaired venous hypertension and venous return resulting from CVD during pregnancy created an environment of cellular hypoxia with associated cell damage, producing extracellular matrix remodelling [10,11,12]. In the present study, we saw an increase in the proangiogenic and lymphangiogenic markers CD31, D2-40, Flt-1, and PIGF in placental villi of women with CVD during pregnancy.

CD31 is altered in placental vascular pathology [24,25]. In an arterial pathology such as preeclampsia, CD31 immunoreactivity is lower in the placenta than in control pregnancies [24,26]. However, Li et al. [27] showed no significant differences in CD31 level in the placenta of women with preeclampsia. In general, angiogenesis and lymphangiogenesis are modified in vascular pathology [28]. Our results show greater CD31 mRNA levels in placental villi of women with CVD, along with more CD31-positive vessels. Podoplanin (D2-40) was also higher in these patients. Our results are consistent with the results of Kandemir et al. [29], showing that podoplanin was increased in the placenta of women with vascular disorders. It should be noted that podoplanin, a mucin-like transmembrane protein, is a specific marker of lymphatic endothelium [30]. The human placenta does not have a mature lymphatic system, but chorionic villous stromal cells express podoplanin [31]. Podoplanin-expressing stromal cells can function as primitive lymphatic vessels [32]. Podoplanin plays a role in foetal angiogenesis during placental development, and abnormal activity leads to pregnancy diseases associated with defective angiogenesis [29,32,33]. Podoplanin expression in the human placenta has been suggested as a response to hypoxic-ischaemic conditions [29]. Placental villi of women with CVD have shown increased expression of hypoxia markers, such as hypoxia-inducible factor-1α [10]. This point would explain why placental villi of women with CVD show higher expression of CD31 and podoplanin, given the cell hypoxia described above.

Vascular abnormalities during pregnancy change angiogenic markers such as Flt-1 and PIGF [34]. These markers and their ratio are important in clinical monitoring of patients with preeclampsia, and they are used on a regular basis [35,36,37]. The Flt-1/PIGF ratio is increasingly used in the study of restricted intrauterine growth [38]. All these pathological events are associated with hypoxic processes affecting placental vascularisation [19]. Placental blood supply is associated with abnormal expression of VEGF and PIGF, and thus of Flt-1 [39]. The gene and protein expression of these markers is abnormal in placental vascular diseases [40]. We also saw this abnormal profile of Flt-1 and PIGF in placental villi, both of which were upregulated in the extracellular matrix. Placental villi of women with CVD have shown an increased expression of VEGF [12], explaining how vascular compromise resulting from CVD during pregnancy induces angiogenesis and lymphangiogenesis in placental villi. The placentas of our women with CVD had more villi with PAS-positive material than the control placentas. The accumulation of polysaccharides suggests these villi are not fully functional, though the processes described aim to correct the situation created by CVD.

## 4. Material and Methods

### 4.1. Study Population

An observational, analytical, and prospective cohort study was conducted on 114 women in their third trimester of pregnancy (32 weeks). Sixty-two were clinically diagnosed with CVD, with a median age of 33 years (22–40) and median gestational age (weeks) of 40.5 (39–41.5). In parallel, 52 controls with no history of CVD (HC) were studied, with a median age of 34 years (27–41) and median gestational age (weeks) of 41 (39–42). This study was conducted according to the basic ethical principles of autonomy, beneficence, non-maleficence, and distributive justice. The study was developed according to the standards of Good Clinical Research Practice, the principles set forth in the last Declaration of Helsinki (2013), and the Oviedo Convention (1997). Patients were informed of the details of the study, and each provided signed consent. The project FIS-PI18/00912 was approved (March of 2017) by the Clinical Research Ethics Committee of the Gómez-Ulla-UAH Defence Hospital (37/17).

Clinical history was picked up during the third-trimester visit, and a general physical exam was carried out. Lower extremities were studied using Doppler ultrasound at 7.5 MHz (Portable M-Turbo Doppler Ultrasound, SonoSite, Inc., Washington, USA). In this work, women over 18 years during the third trimester of pregnancy with clinical evidence of lower extremity CVD were included, with clinical, etiological, anatomical, pathological (CEAP) C-classes ≥1 (C1 = 59.67% (n = 37), C2 = 33.87% (n = 21), C3 = 6.45% (n = 4)). Examination of the venous system of the lower extremities of the study patients was performed using a 7.5 MHz Eco-Doppler transducer (M-Turbo Eco-Doppler, SonoSite, Inc.). A detailed examination (main saphenous axis from the inguinal region to the ankle and femoral veins) was carried out with the woman standing and her leg in external rotation. Exclusion criteria were defined by women with endocrine diseases as diabetes mellitus; high blood pressure (HBP); body mass index (BMI) ≥ 25 kg/m²; unhealthy habits; active infectious diseases; autoimmune diseases; venous malformations; kidney, heart, or lung failure; preeclampsia and/or haemolysis, elevated liver enzymes and low platelets (HELLP) syndrome; intrauterine growth restriction by known causes; presence of pathological lesions such as placental infarcts, avascular villi, delayed maturation, and chronic inflammation affecting chorionic villi, along with the emergence of any of the previous exclusion criteria any time before to delivery; as well as prior evidence of CVD.

### 4.2. Placental Tissue Samples

After delivery, placental biopsies were collected. To ensure that the samples included multiple cotyledons, five placental pieces were obtained using a scalpel in all cases. Placental fragments were put into two different sterile tubes—one including minimum essential medium (MEM) with 1% antibiotic/antimycotic (both from ThermoFisher Scientific, Waltham, MA, USA) and another containing RNAlater^®^ solution (Ambion, Austin, TX, USA). In the laboratory, the samples were processed in a sterile environment in a class II laminar flow hood (Telstar AV 30/70 Müller 220 V 50 MHz; Telstar SA Group, Terrassa, Spain). Samples were kept in 1 mL of RNAlater^®^ at –80°C and preserved until further processing for gene expression analysis. MEM samples were destined to histological and immunodetection studies.

Samples within MEM were washed and rehydrated various times with antibiotic-free medium, eliminating blood cells, then divided into fragments, and afterwards fixed in F13 (60% ethanol, 20% methanol, 7% polyethylene glycol, 13% distilled H_2_O), as shown in established protocols. Following inclusion, paraffin blocks were made using molds. Posterior to paraffin’s solidification, an HM 350 S rotation microtome (Thermo Fisher Scientific, MA, USA) was used to produce 5 µm thick sections, which were taken to a hot water bath, collecting them on a glass slides, and treated with 10% polylysine for better adhesion of the cuts.

### 4.3. Gene Expression Analysis Using RT-qPCR

RNA extraction was executed according to the guanidinium thiocyanate-phenol-chloroform method described by Ortega et al. [41]. RT-qPCR was performed in a StepOnePlus™ System (Applied Biosystems – Life Technologies, Waltham, MA, USA), with the standard curve method. The reaction was carried out as follows: 5 µL of each sample in a 1:20 dilution in nuclease-free water was mixed with 10 µL of DNase- and RNase-free water and then placed into a MicroAmp^®^ 96-well plate (Applied Biosystems – Life Techno logies) for a final reaction volume of 20 µL. All sequences were designed de novo as follows in Table 1, using the Primer-BLAST [42].

### 4.4. Protein Expression Analysis by Immunohistochemistry

Detection of the antigen–antibody reaction was achieved by using the avidin–biotin complex (ABC) method, with peroxidase or alkaline phosphatase as chromogens. The whole procedure was described meticulously by Ortega et al. [43]. Firstly, samples were incubated at 4 °C overnight in a dilution composed by primary antibody, BSA 3%, and PBS, as detailed in Table 2A. Afterwards, samples were incubated in biotin-conjugated secondary antibody, and diluted in PBS during 1.5 h at room temperature (Table 2B). Finally, the avidin–peroxidase conjugate ExtrAvidin^®^–Peroxidase (Sigma-Aldrich, St. Louis, MO, USA) was applied for 60 min at room temperature (1:200 dilution in PBS), using the chromogenic substrate diaminobenzidine (Kit DAB, SK-4100, Vector Laboratories, Burlingame, CA, USA) to reveal, which was prepared just before exposure (5 mL of distilled water, two drops of buffer, four drops of DAB, two drops of hydrogen peroxide), eventually resulting in a brown stain. In all immunohistochemical studies, sections of the same tissue were used as a negative control, in which primary antibody incubation was substituted with incubation in blocking solution (PBS).

### 4.5. Periodic Acid of Schiff (PAS) Staining

PAS staining is one of the most common methods used in histology to show the presence of aldehyde groups derived from carbohydrate oxidation. We used a PAS staining kit (cat. #1016460001) from MERCK (Darmstadt, Germany). Once the sections were deparaffinized and rehydrated, they were rinsed with distilled water. The histological material was first treated with periodic acid for 5 min to oxidize 1,2-glycols to aldehyde groups. To obtain a high and suitable staining specificity, the tissue was treated with sulfite water (3 × 2 min) after the reaction with periodic acid. Sulfite water was prepared by first mixing 10 mL of 10% sodium bisulfite solution and 10 mL of 1 mol/L hydrochloric acid, and then this solution was mixed with 200 mL of tap water. By adding the Schiff reagent (leucofuchsin) in the second step (15 min), aldehydes reacted, producing an intense red colour. To obtain a bright and contrasting pattern of PAS-positive structures, a haematoxylin solution modified according to Gill II was used. Deparaffinization, rehydration, and mounting processes were as usual [44].

### 4.6. Statistical Analysis and Interpretation of Results

The statistical analysis was carried out using the program GraphPad Prism^®^ 6.0. The Mann–Whitney U and Pearson χ² tests were studied in this work. The obtained data are expressed as the median with interquartile range (IQR) and significance was established at *p* < 0.05 (*), *p* < 0.01 (**), and *p* < 0.001 (***). Five sections and ten fields per section were randomly analyzed for each patient classified in their established groups. Patients were considered positive when the marked average area in the analysed sample was ≥5% of the total, following the pathology protocol. The slides were examined under a Zeiss Axiophot optical microscope (Carl Zeiss, Germany).

## 5. Conclusions

Our results show for the first time how, in pregnant women with CVD, normal vascularization of villi is affected in placental villi. These villi have upregulated angiogenesis and lymphangiogenesis markers, related to an increase in vascularisation. It is necessary to know how these events can compromise the function of placental villi and, therefore, the maternal–foetal gas exchange.

## Figures and Tables

**Table 1:** RT-qPCR primer sequences and temperature (Temp). PIGF, placental growth factor.

GENE	SEQUENCE Fwd (5′→3′)	SEQUENCE Rev (5′→3′)	Temp
**TBP**	TGCACAGGAGCCAAGAGTGAA	CACATCACAGCTCCCCACCA	60 °C
**CD-31**	ACTGCACAGCCTTCAACAGA	TTCTTCCATGGGGCAAG	60 °C
**D2-40**	TGACTCCAGGAACCAGCGAAG	GCGAATGCCTGTTACACTGTTGA	50 °C
**Flt-1**	TCAGCTACTGGGACACCGG	CCTGAACTAGATCCTGTGAGAAGCA	60 °C
**PIGF**	CAGAGGTGGAAGTGGTACCCTTCC	CGGATCTTTAGGAGCTGCATGGTGAC	58 °C

**Table 2:** Antibodies (A. primary, B. secondary) used in the immunohistochemical studies performed with dilutions and specifications protocols.

**A**				
**Antigen**	**Species**	**Dilution**	**Provider**	**Protocol specifications**
CD-31	Mouse monoclonal	1:100	P-8590 (Sigma-Aldrich)	
D2-40	Mouse monoclonal	1:100	M3619 (Agilent, Dako)	
Flt-1	Mouse monoclonal	1:500	sc-271789 (Santa cruz biotechnology)	Citrate tampon in heat (pH =6)
PlGF	Rabbit polyclonal	1:250	ab230516 (Abcam)	EDTA pH = 9 before incubation with blocking solution
**B**				
**Antigen**	**Species**	**Dilution**	**Provider**	**Protocol specifications**
IgG (Mouse)	Goat polyclonal	1:300	Sigma-Aldrich (F2012/045K6072 )	
IgG (Rabbit)	Mouse polyclonal	1:1000	Sigma-Aldrich (RG-96/ B5283)	
